# Ultrasonic humidifier lung as a mimic of COVID‐19

**DOI:** 10.1002/rcr2.761

**Published:** 2021-05-04

**Authors:** Shosei Ro, Ryosuke Imai, Atsushi Kitamura, Torahiko Jinta, Naoki Nishimura

**Affiliations:** ^1^ Department of Pulmonary Medicine Thoracic Center, St. Luke's International Hospital Chuo‐ku Japan

**Keywords:** Chest computed tomography, COVID‐19, ultrasonic humidifier lung

## Abstract

Chest computed tomography (CT) has been used to complement coronavirus disease 2019 (COVID‐19) diagnosis due to its high sensitivity. However, owing to the low specificity of CT findings, differential diagnosis is essential. The typical CT findings of COVID‐19 include ground‐glass opacifications and consolidations with predominant distribution in bilateral, peripheral, and subpleural parts of the lung. These imaging findings are non‐specific and may resemble other lung conditions, including ultrasonic humidifier lung, which is a condition that develops on inhaling aerosols generated by ultrasonic humidifiers. We present two patients with initial symptoms similar to COVID‐19. CT examination revealed centrilobular nodules and consolidations with upper lobe‐predominant distribution, although atypical for COVID‐19, but key findings for ultrasonic humidifier lung. Therefore, ultrasonic humidifier lung could be a differential diagnosis for COVID‐19 in dry environments. Characteristic CT findings and a history of ultrasonic humidifier use are critical to the final diagnosis.

## Graphical abstract

We report two cases of patients diagnosed with ultrasonic humidifier lung with characteristic computed tomography (CT) findings of centrilobular nodules and consolidations with upper lobe‐predominant distribution, who were initially suspected of coronavirus disease 2019 (COVID‐19) infection.

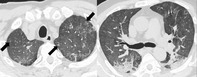

## Introduction

Severe acute respiratory syndrome coronavirus 2 (SARS‐CoV‐2) polymerase chain reaction (PCR) testing is considered the gold standard in the diagnosis of coronavirus disease 2019 (COVID‐19), and chest computed tomography (CT) has been used to complement this modality during the pandemic. However, various other lung diseases could mimic the CT findings of COVID‐19, making differential diagnosis essential [1]. To our knowledge, there is no report on differentiating ultrasonic humidifier lung from COVID‐19. We report the cases of two patients who were initially suspected of COVID‐19 infection but were diagnosed with ultrasonic humidifier lung with characteristic CT findings of centrilobular nodules and consolidations with upper lobe‐predominant distribution.

## Case Report

### Case 1

**Figure 1:**
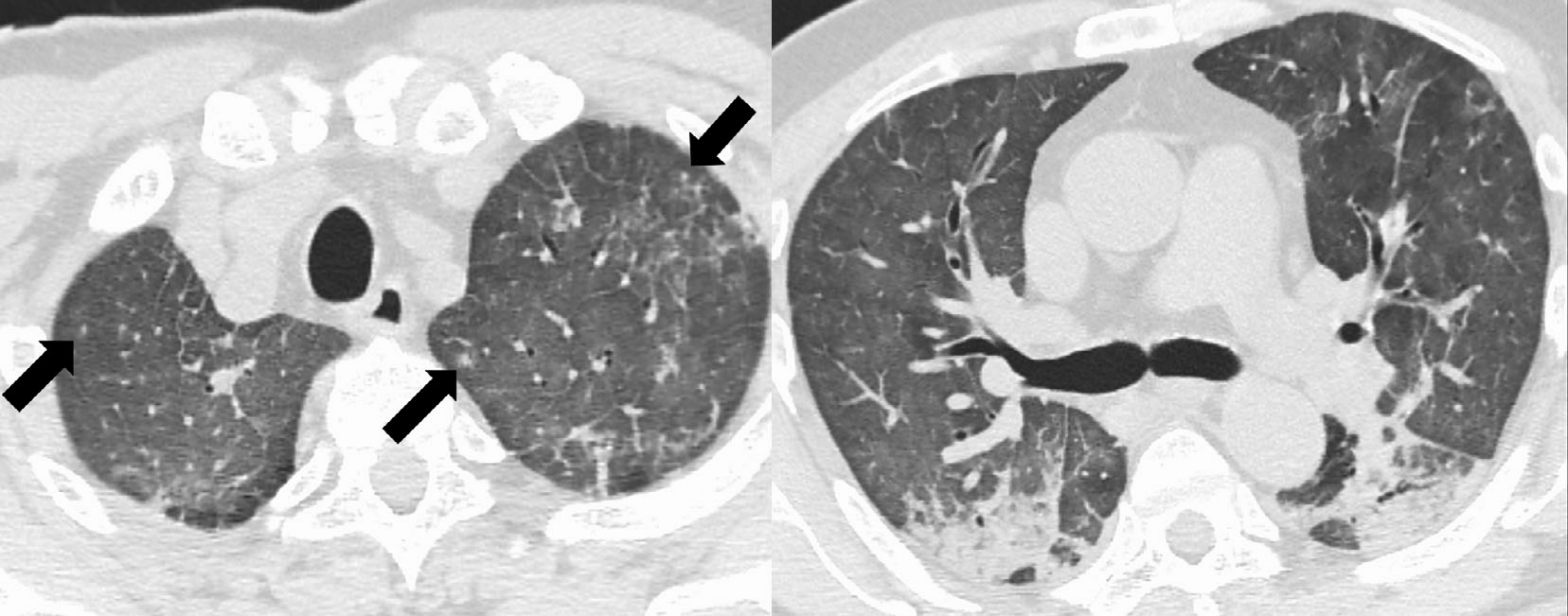
Chest computed tomography (CT) in case 1 showed centrilobular particles (see arrows). Consolidations were seen on both lungs' dorsal area, which was similar to those found in coronavirus disease 2019 (COVID‐19).

A 64‐year‐old man presented to our hospital with a four‐week history of fever and dyspnoea. An oxygen saturation level of 88% was obtained with the oxygen mask at 10 L/min. He was intubated, and mechanical ventilation was initiated. Laboratory data demonstrated that the white blood cell count (WBC), lactate dehydrogenase (LDH), C‐reactive protein (CRP), and Krebs von den Lungen‐6 (KL‐6) levels were 10,200/μL, 366 U/L, 17.52 mg/dL, and 416 U/mL, respectively. CT showed centrilobular nodules with bilateral diffuse ground‐glass opacifications (GGO) and consolidations in the dorsal areas of both lungs (Fig. 1). We initially suspected COVID‐19. Because of severe respiratory failure, methylprednisolone (1000 mg/day for three days) for lung injury and empiric piperacillin and tazobactam (13.5 g/day) for suspected lung infection were initiated before the diagnosis was confirmed. However, repeated PCR and blood and sputum cultures showed negative results. The medical interview revealed that the patient had been using an ultrasonic humidifier without changing its water or cleaning the tanks for a month before admission. We suspected ultrasonic humidifier lung based on the history and CT findings. The fever and GGO lesions on chest radiographs improved within three days. Based on this characteristic history and hospital course, we finally diagnosed the ultrasonic humidifier lung. The patient's condition and the CT findings resolved on avoiding humidifier use after discharge.

### Case 2

**Figure 2:**
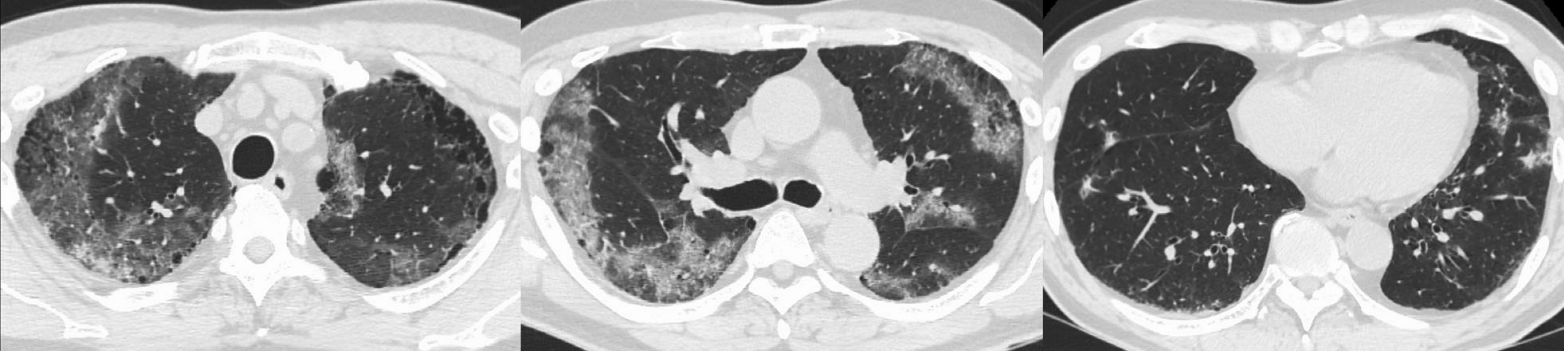
Chest computed tomography (CT) in case 2 showed peripheral consolidation predominant in the upper lobes.

A 62‐year‐old man with a one‐week history of fever, dyspnoea, and throat discomfort was admitted to our hospital. Laboratory data showed that the levels of the WBC count, LDH, CRP, and KL‐6 were 8300/μL, 290 U/L, 9.67 mg/dL, and 570 U/mL, respectively. Chest CT showed non‐segmental GGO and peripheral linear consolidations predominantly on both upper lobes (Fig. 2). We strongly suspected COVID‐19. However, repeated PCR and blood and sputum cultures showed negative results. The patient had a history of ultrasonic humidifier use, and had not changed the water or cleaned the tanks for more than a month before admission. The patient was finally diagnosed with ultrasonic humidifier lung. After antigen avoidance, his condition resolved with improved chest CT findings and he has not experienced any symptom relapse without pharmacological therapies.

## Discussion

Here, we reported two patients diagnosed with ultrasonic humidifier lung who were initially suspected of having COVID‐19. The CT findings of centrilobular nodules and consolidations with upper lobe‐predominant distribution and a history of humidifier usage were vital to the diagnosis.

The humidifier lung was first reported in the 1970s as a phenotype of hypersensitivity pneumonitis associated with a large‐scale humidifier system and was mainly considered an occupational disease until recently [2]. The incidence rate is approximately 10% among exposed people [3]. In Japan, 4.3% of hypersensitivity pneumonitis was reported to be humidifier lung, which may have increased in recent years with the wide use of domestic ultrasonic humidifiers [4, 5]. A seasonal increase in the cases of humidifier lung has been reported in the winter months of December to February in Japan, when ultrasonic humidifiers are used to prevent dry indoor environments [6]. Fever, cough, and dyspnoea are common symptoms [3]. Microorganisms such as fungi and bacteria grow inside the ultrasonic humidifiers when the water is not replaced, or the tank is not cleaned regularly. Inhaling droplets containing them can cause ultrasonic humidifier lung. Many microorganisms have been reported to cause allergic reactions that can lead to humidifier lung. Endotoxin in the highly contaminated water in the humidifier tank is suggested to induce severe lung injury [5]. Therefore, antibiotic agents are normally ineffective. Cessation from humidifier use is most important to avoid antigens. Corticosteroids may also be effective in severe lung injury [2, 6].

The two patients of this study were finally diagnosed as humidifier lung, and their CT findings were similar to COVID‐19. Although PCR is considered the gold standard in the diagnosis of COVID‐19, chest CT is used to complement the diagnosis in endemic areas, owing to its high sensitivity (97%) [7]. Typical CT findings of COVID‐19 include the presence of GGOs alone (50.2%) and with consolidations (44.2%), with predominant distribution in bilateral, peripheral/subpleural, and lower lobes [8]. However, CT has low specificity (25%), and therefore, it is important to differentiate COVID‐19 from other diseases with similar CT findings. CT findings of ultrasonic humidifier lung are characterized by GGO (88.9%), centrilobular nodules (27.8%), peribronchovascular or subpleural nonsegmental consolidations (44.4%), mosaic attenuation (50.0%), and subpleural curve linear opacity (11.1%). Unlike summer‐type hypersensitivity pneumonitis, which is the most common phenotype of hypersensitivity pneumonitis in Japan, it is characterized not only by GGO or centrilobular nodules but also by subpleural consolidations. These findings are the result of significant inflammation induced by direct inhalation of aerosolized bacteria or their derived endotoxin [6]. Both patients presented with fever and dyspnoea during the COVID‐19 pandemic, with CT findings of GGO and subpleural consolidation, initially suggesting COVID‐19. However, the CT findings of centrilobular nodules with upper lobe‐predominant distribution were atypical for COVID‐19. A review of the CT findings of COVID‐19 showed typically a lack of centrilobular changes with lower lobe‐predominant distribution, unlike other diseases [1].

Long‐term data such as pulmonary function tests and 6‐min walk tests were not obtained because of the limitation in performing such examinations in the pandemic. However, our patients returned to daily life without dyspnoea, and chest radiographs showed improvement. In addition, challenge tests and bronchoscopy were not performed considering both patients' non‐compliance and initial respiratory status. The diagnosis of ultrasonic humidifier lung was based on the respiratory symptoms lasting for more than one week, typical CT findings of bilateral GGO and consolidations, history of humidifier usage, and improvement in the patients' condition on avoiding humidifier use [5, 6].

Ultrasonic humidifier lung is not well recognized and may be underestimated. To our knowledge, there is no report on differentiating ultrasonic humidifier lung from COVID‐19. Characteristic CT findings and a history of ultrasonic humidifier use are critical in making this distinction.

### Disclosure Statement

Appropriate written informed consent was obtained for publication of this case report and accompanying images.

### Author Contribution Statement

All authors met the authorship criteria. Shosei Ro wrote the manuscript. Ryosuke Imai, Atsushi Kitamura, Torahiko Jinta, and Naoki Nishimura revised and edited the manuscript. All the authors approved the final version of the manuscript to be published.
